# Handgrip weakness and overall life satisfaction decline: derivation of cutoff values and analysis of sex differences in older Chinese adults

**DOI:** 10.3389/fnut.2025.1537818

**Published:** 2025-03-18

**Authors:** Liangyu Yin, Lan Zhong

**Affiliations:** ^1^Department of Nephrology, The Key Laboratory for the Prevention and Treatment of Chronic Kidney Disease of Chongqing, Chongqing Clinical Research Center of Kidney and Urology Diseases, Xinqiao Hospital, Army Medical University (Third Military Medical University), Chongqing, China; ^2^Department of Clinical Nutrition, The People’s Hospital of Liangping District, Chongqing, China

**Keywords:** handgrip strength, life satisfaction, cutoff values, older adults, sex difference

## Abstract

**Background and aims:**

Handgrip strength (HGS) is a cost-effective indicator of skeletal muscle function. However, the sex-specific association between HGS and life satisfaction decline among older Chinese adults remains largely unknown.

**Methods:**

This observational, cross-sectional multicenter study included 3,649 older adults (age range: 60–101 years) from a nationally representative survey in China. Overall life satisfaction was determined using a life satisfaction score (LSS). Correlations between variables were examined using a Spearman’s correlation analysis. Receiver operating characteristic (ROC) curves were utilized to determine the HGS cutoffs for predicting a decline in LSS. Restricted cubic spline (RCS) analysis and multivariate logistic regression were employed to investigate the associations between low HGS and LSS.

**Results:**

This study included 1,762 women and 1,887 men (median age = 68.3 years). LSS decline was observed in 485 (13.3%) participants. HGS was positively correlated with LSS in both men and women (both *P* < 0.05). Individuals with low HGS were associated with a higher rate of LSS decline (16.2% vs. 10.8%, *P* < 0.001). RCS analysis demonstrated a linear-like association between HGS and life satisfaction in men (*P* < 0.001, *P* nonlinear = 0.099), but not in women (*P* = 0.110, *P* nonlinear = 0.329). ROC analysis revealed that the optimal HGS cutoff for indicating the presence of LSS was 27.5 kg for men and 22.3 kg for women. Multivariable analysis showed that participants with low HGS had higher odds of experiencing a decline in LSS [odds ratios (OR) = 1.509, 95% confidence interval (CI) = 1.218–1.867]. This association was observed only in men (OR = 1.871, 95% CI = 1.358–2.562, *P* < 0.001), while it was attenuated in women (OR = 1.281, 95% CI = 0.964–1.701, *P* = 0.087).

**Conclusion:**

This study establishes sex-specific cutoffs of HGS for identifying a decline in LSS among older Chinese adults. Low HGS is positively associated with LSS decline among men in a linear-like manner, but not among women. These findings might facilitate the development of strategies to promote healthy aging.

## Introduction

Aging has recently become a significant focus of medical and public health concerns worldwide (1). China, in particular, boasts the largest older adults globally, projected to reach 479 million individuals aged 60 and above by 2050 (2). As individuals age, their muscle strength gradually diminishes, potentially reaching a point where weakness hampers their ability to carry out daily activities effectively (3). Exercise training that focuses on improving muscle strength and/or function has demonstrated functional benefits for both healthy adults (4) and patients (5).

Handgrip strength (HGS) is a measure of muscle strength and function in the hand and forearm muscles. The procedures used to measure HGS are convenient, safe, and reliable, and do not require large or expensive equipment (6). These characteristics make HGS a cost-effective indicator of overall skeletal muscle strength in epidemiological studies and clinical practice (7, 8). Notably, both the European Working Group on Sarcopenia in Older People (EWGSOP) (9) and the Asian Working Group for Sarcopenia (AWGS) (10) have included HGS as the sole indicator of muscle strength in their latest guidelines for diagnosing sarcopenia. Additionally, low HGS has been linked to various health risks, including Parkinson’ s disease (11), hypertension (12), metabolic syndrome (13), diabetes (14), as well as all-cause mortality (15–18) and cause-specific mortality from respiratory disease (17), cardiovascular disease (16, 18), and cancer (17–19). Other studies have also shown that HGS is associated with the maintenance of cognitive function (20) and healthy aging (21) in community-dwelling older adults. These findings support HGS as a simple and effective diagnostic and prognostic indicator for health purposes.

Despite these lines of evidence, the impact of HGS on quality of life (QoL) remains controversial. A recent study involving patients with colorectal cancer suggests a positive correlation between HGS and health-related QoL (22). However, another study with a similar design found no significant association (23). In Asian oncology patients, a study revealed that maintaining or improving HGS through regular exercise is crucial for the QoL of older adults who survived from cancer (24). Conversely, in older individuals with Alzheimer’s disease, no significant relationship was found between HGS and QoL (25). The association between HGS and self-reported life satisfaction in older Chinese adults living in communities has not been thoroughly studied. Additionally, HGS can vary depending on factors such as sex (26), ethnic background (9, 10), diseases (19, 27), and even the type of dynamometer used (6). Thus, it is crucial to establish specific cutoff values that can be adjusted for different scenarios and demographic groups, enhancing the practicality of HGS measurement.

The China Health and Retirement Longitudinal Study (CHARLS) is an ongoing nationwide survey in China (28). In 2011, the CHARLS project recruited participants from 10,257 households spanning 150 counties or districts and 450 villages across 28 provinces in China. This comprehensive coverage of diverse regions makes CHARLS an ideal data source for establishing cutoff values applicable to older adults residing in communities. The objective of this study was to determine sex-specific, life satisfaction-oriented cutoff points for low HGS in older Chinese adults based on data obtained from CHARLS. Specifically, we examined the association between handgrip weakness and life satisfaction, considering factors such as age, gender, and other relevant variables, to estimate potential underlying effects and modifications.

## Materials and methods

### Study design and population

This was a cross-sectional study. Study participants were enrolled from an ongoing nationally representative longitudinal survey in China, the CHARLS (29). The CHARLS project employs a structured questionnaire to collect high-quality data through in-person interviews from a nationally representative sample of Chinese adults aged 45 years and older. The questionnaire includes standardized assessments of sociodemographic and lifestyle factors, as well as health-related information. In 2011, the CHARLS study recruited participants from 10,257 households across 28 provinces in China. The collected data were weighted to ensure that the survey sample accurately represented the national population. For more comprehensive information about the study design of CHARLS, refer to previous publications (28).

**FIGURE 1:**
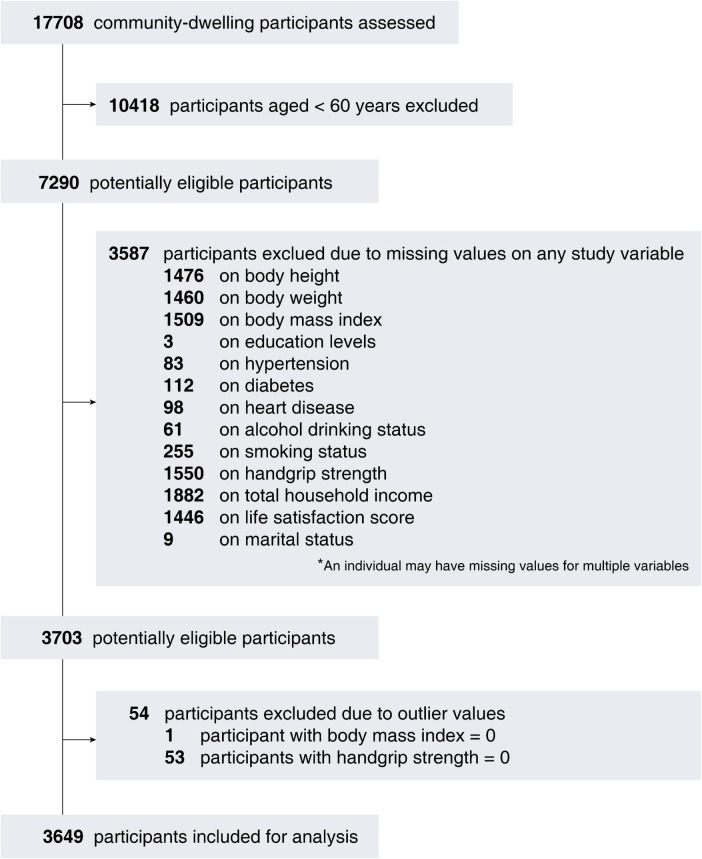
A flowchart of the subject inclusion.

In this study, we conducted a retrospective analysis of data from the CHARLS surveys conducted in 2011. The inclusion criteria were as follows: (1) individuals aged 60 and above at baseline; and (2) those with available data on HGS and life satisfaction score (LSS). Exclusion criteria included: (1) individuals aged <60 years upon recruitment; (2) missing data on HGS and LSS at baseline; (3) missing data on relevant study variables; and (4) participants with outlier values. A flowchart illustrating the inclusion of subjects is provided in Figure 1. Among the 17,708 baseline subjects initially studied, 10,418 subjects aged <60 years were excluded. Additionally, 3,587 individuals were excluded due to missing key study variables necessary for analysis. Further exclusion was made for 54 subjects with suspected outlier values. This left 3,649 individuals for formal analysis (age range: 60–101 years). The research protocol received approval from an institutional Ethical Review Committee (approval number: IRB00001052-11015), and all participants provided informed consent. The procedures involving human participants adhered to the principles outlined in the 1964 Helsinki Declaration and its subsequent amendments, or equivalent ethical standards, as well as the ethical standards set by the institutional and/or national research committee. This study was conducted following the Strengthening the Reporting of Observational Studies in Epidemiology (STROBE) guidelines.

### Exposure: handgrip strength

Handgrip strength (kg) was measured using a dynamometer (Model: Yuejian™ WL-1000, Nantong Yuejian Physical Measurement Instrument Co., Ltd., Nantong, China) (28). Individuals were instructed to perform a maximal isometric contraction with their dominant hand at a 90° angle to obtain the HGS value while standing. Measurements were performed two consecutive times and the maximal value was used for analysis. Each effort lasted several seconds. Receiver operating characteristic (ROC) curves were utilized to determine cutoffs for HGS in predicting the decline in LSS. Area under the curve (AUC) along with a 95% confidence interval (CI), sensitivity, specificity, positive predictive value (PPV), and negative predictive value (NPV) were used to quantify the performance of HGS to predict LSS decline. Weight-adjusted HGS (defined as HGS in kg divided by body weight in kg, HGS/weight) and body mass index (BMI)-adjusted HGS (defined as HGS in kg divided by BMI in kg/m^2^, HGS/BMI) were created. Their performance in predicting life satisfaction decline was statistically compared to HGS using DeLong’s test.

### Outcome: life satisfaction score

The main outcome of the present study was defined as a considerable decline in the LSS. LSS reflects participants’ self-reported level of life satisfaction. Within the CHARLS survey, life satisfaction is assessed with the following question: “Please think about your life-as-a-whole. How satisfied are you with it?” Responses to this question were categorized as follows: (1) Not at all satisfied; (2) Not very satisfied; (3) Somewhat satisfied; (4) Very satisfied; and (5) Completely satisfied. The continuous score was dichotomized into a binary variable for subsequent statistical analysis: not very satisfied or worse vs. somewhat satisfied or better. Participants reporting not very satisfied or worse LSS were identified as experiencing a decline in LSS.

### Covariates

The baseline characteristics of the study population were obtained by trained researchers using a structured questionnaire on socio-demographic status and health-related factors. Social-demographic factors included age, sex, residency (rural vs. urban), household income and marital status (married vs. other), and education level (elementary school and below vs. secondary school/college and above). Health-related factors included BMI, drinking status (yes vs. no), smoking (yes vs. no), and comorbidities (hypertension, diabetes, heart disease). History of comorbidities including hypertension, diabetes, and heart diseases were acquired with the following question: “Have you been diagnosed by a doctor with conditions listed below?” BMI was also categorized as underweight (<18.5), normal (18.5 to <24), overweight (24 to <28), or obese (≥28) based on Chinese recommendations (30).

### Statistical analysis

Continuous data were presented as medians [25th percentile, 75th percentile] and were compared using a nonparametric Wilcoxon’s rank-sum test. Categorical data were expressed as numbers (percentages) and compared using a Chi-squared test. For the baseline analysis, all baseline characteristics, excluding BMI, were weighted at individual level with household and response adjustment. BMI is weighted by biomarker individual level with household and response adjustment (2). Other analyses were conducted without weighting (2).

The correlation between two variables was assessed using Spearman’s rank correlation analysis and visualized through an integrated correlation matrix and heat map of correlation coefficients. The potential nonlinear relationship between continuous HGS values and LSS decline in different sexes was flexibly analyzed using the restricted cubic spline (RCS) method. Nonlinearity was statistically tested using a likelihood ratio test, with a significance level set at *P* < 0.05.

The associations between HGS and LSS decline were evaluated using multivariable logistic regressions. The HGS was analyzed as a continuous variable, per each standard deviation and as a categorical variable. Odds ratios (OR) with 95% CI were calculated to estimate the effects. Incremental models were developed with increasing numbers of covariates: model 0 as the unadjusted crude model, model 1 adjusted for age at baseline and sex, model 2 adjusted for age at baseline, sex, BMI, education level, hypertension, diabetes, and heart disease. Model 3, the fully adjusted model, included all variables in model 3 plus alcohol drinking, smoking, residency, total household income, and marital status. In an exploratory logistic regression analysis, we selected a subset of the study population (Supplementary Figure 1) to allow further adjustment for vigorous, moderate, and mild physical activity (defined as at least 10 min of vigorous, moderate, or mild energetic physical activity per week, respectively), as well as sleep duration (defined as the average number of hours slept per night over the past month).

Subgroup and interaction analyses were performed in different strata of the adjusting variables to evaluate modification of the associations observed in the overall population and to determine the applicability of HGS to predict LSS decline across different subgroups. These analyses were based on age (<70 vs. ≥70 years) (31), sex (women vs. men), BMI categories (underweight, normal, overweight, and obese), educational level (lower vs. higher), hypertension (yes vs. no), diabetes (yes vs. no), heart disease (yes vs. no), residence (rural vs. urban), drinking status (yes vs. no), smoking status (yes vs. no), total household income (<10,000 CNY vs. ≥10,000 CNY), and marital status (married vs. other). Multiplicative interactions were tested by adjusting the cross-product terms of the HGS and other covariates. Covariates showing statistically significant multiplicative interactions (*P* < 0.10) were considered potential effect modifiers. All tests were two-sided, and significance was set at *P* < 0.05 unless otherwise specified. The analyses were conducted using R (version 4.3.1, Foundation for Statistical Computing, Vienna, Austria).

## Results

### Subject inclusion and overview of the study sample

The study sample comprised of 1,762 women and 1,887 men, with a median age of 68.3 years. There were 485 participants who reported a declined LSS according to the study’s definition. Other baseline characteristics of the study population are shown in the overall column of Table 1.

**TABLE 1:** Weighted baseline characteristics of the study population by handgrip strength groups.

		Handgrip strength		
**Characteristics**	**All (*n* = 3,649)**	**Normal (*n* = 2,746)**	**Low (*n* = 1,173)**	***P***
Age, years	68.3 (6.6)^1^	67.1 (5.8)	70.9 (7.3)	<0.001
Age, ≥70 years	36.9^2^	29.1	52.6	<0.001
Sex, man	51.3	60.9	31.9	<0.001
Residency, rural village	56.5	55.5	58.6	0.170
Total household income, 10,000 CNY	2.5 (3.2)	2.7 (3.3)	2.0 (3.0)	<0.001
Total household income, ≥10,000 CNY	54.4	58	47.1	<0.001
Education level, secondary school/college and above	4.9	6.1	2.5	<0.001
Measured body mass index, kg/m^2^	24.4 (42.1)	25.4 (51.3)	22.4 (4.3)	0.046
Measured body mass index, group				<0.001
Underweight	9.8	7.3	14.9	
Normal	53.3	52.6	54.8	
Overweight	26.1	27.8	22.5	
Obese	10.8	12.2	7.8	
Alcohol drinking, yes	30.8	35.4	21.4	<0.001
Smoking, yes	30.6	33.8	24.1	<0.001
Marital status, unmarried	27.0	21.5	38.2	<0.001
Comorbidities				
Hypertension, yes	35.5	35.2	36.1	0.672
Diabetes, yes	8.3	8.1	8.8	0.576
Heart disease, yes	17.3	17.6	16.8	0.646
Satisfaction of life score				0.001
1 = Not at all satisfied	2.1	1.7	3.1	
2 = Not very satisfied	10.5	9.1	13.2	
3 = Somewhat satisfied	62.1	63	60.3	
4 = Very satisfied	23.1	23.7	22	
5 = Completely satisfied	2.1	2.5	1.4	
Declined satisfaction of life score, <3	12.6	10.8	16.2	<0.001
Handgrip strength, kg	29.0 (10.1)	34.0 (7.9)	18.7 (5.0)	<0.001

CNY, Chinese Yuan.

^1^Mean (SD), all such values, compared using a Student’s *t*-test.

^2^Percentage, all such values, compared using a Chi-squared test.

### Optimal handgrip strength variable

The sex-specific performance of different HGS variables in predicting LSS decline was compared. In men, HGS outperformed both HGS/BMI (AUC: 0.576 vs. 0.538, Delong’s *P* = 0.001) and HGS/weight (AUC: 0.576 vs. 0.526, Delong’s *P* < 0.001; Supplementary Figure 2A). In women, HGS demonstrated comparable performance to HGS/BMI (AUC: 0.537 vs. 0.514, Delong’s *P* = 0.174) and HGS/weight (AUC: 0.537 vs. 0.512, Delong’s *P* = 0.086; Supplementary Figure 2B). Therefore, HGS was selected for subsequent analysis.

### Distribution of handgrip strength

**FIGURE 2:**
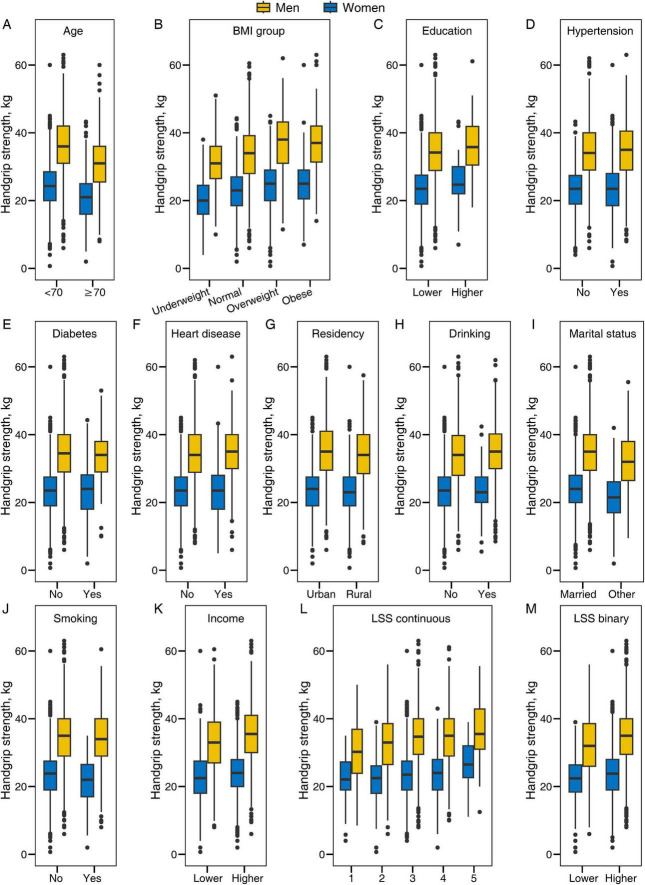
Sex-specific distribution of the handgrip strength stratified by characteristics of participants. BMI, body mass index; LSS, life satisfaction score. **(A)** Age. **(B)** BMI category. **(C)** Education level. **(D)** Hypertension. **(E)** Diabetes. **(F)** Heart disease. **(G)** Residency. **(H)** Drinking. **(I)** Marital status. **(J)** Smoking. **(K)** Income. **(L)** Life satisfaction score (continuous). **(M)** Life satisfaction score (binary).

Figure 2 illustrates the sex-specific distribution of HGS categorized by different baseline characteristics. Overall, men exhibited higher HGS values compared to women. Specifically, participants who were younger, had higher BMI, a higher education level, resided in urban areas, married, had no history of smoking, higher income, and higher LSS tended to have higher HGS values. Moreover, the distribution pattern of HGS appeared to vary by sex across different strata of hypertension, diabetes, heart disease, and alcohol consumption.

### Correlation of the handgrip strength with baseline characteristics

**FIGURE 3:**
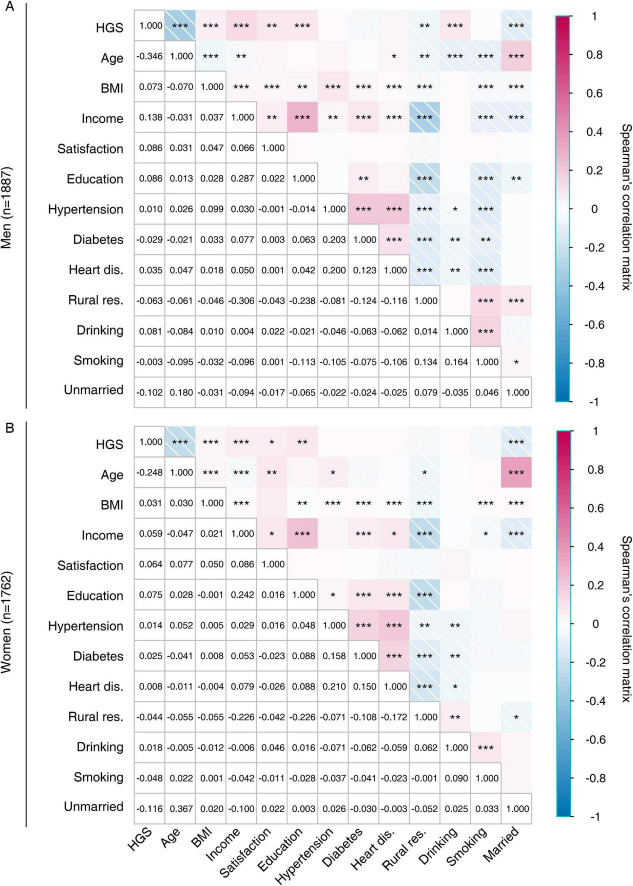
Correlation matrix of the study variables by sex. HGS, handgrip strength; BMI, body mass index; dis., disease; res., residency. *P < 0.05; **P < 0.01; ***P < 0.001. **(A)** Correlation matrix in men. **(B)** Correlation matrix in women.

Sex-specific Spearman’s rank correlation tests were conducted to evaluate the degree of association between continuous HGS and various baseline features (Figure 3). For men, the HGS value showed positive correlations with BMI (*r* = 0.073, *P* < 0.001), income (*r* = 0.138, *P* < 0.001), LSS (*r* = 0.086, *P* = 0.002), education level (*r* = 0.050, *P* = 0.031), and alcohol drinking (*r* = 0.081, *P* = 0.001), while displaying negative correlations with age (*r* = −0.081, *P* < 0.001), rural residency (*r* = −0.063, *P* = 0.010), and unmarried status (*r* = −0.102, *P* < 0.001; Figure 3A). For women, the HGS value exhibited positive correlations with BMI (*r* = 0.031, *P* < 0.001), income (*r* = 0.059, *P* < 0.001), LSS (*r* = 0.064, *P* = 0.011), and education level (*r* = 0.054, *P* = 0.023), but displayed a negative correlation with age (*r* = −0.248, *P* < 0.001) and unmarried status (*r* = −0.116, *P* < 0.001; Figure 3B).

### Restricted cubic spine analysis

**FIGURE 4:**
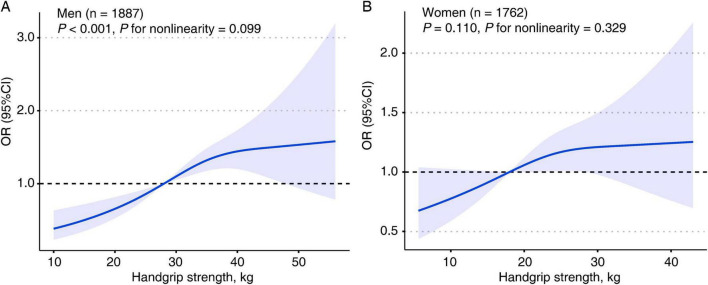
Restricted cubic spine (RCS) analyses on the association between handgrip strength and life satisfaction score. **(A)** RCS analysis in men. **(B)** RCS analysis in women.

Nonlinear associations between HGS and the binary LSS categories (high vs. low) were examined using RCS analysis by sex. In men, the RCS analysis revealed a linear association (*P* < 0.001; Figure 4A) between continuous HGS and increased odds of higher LSS, with no significant nonlinearity (*P* for nonlinearity = 0.099). Conversely, the relationship between HGS and LSS in women was not statistically significant (*P* = 0.110; Figure 4B).

### Cutoff values of handgrip strength to predict decline of life satisfaction score

Sex-specific cutoff values for HGS to predict LSS decline were determined. For men, the optimal cutoff value for HGS was 27.5 kg (sensitivity = 32.6%, specificity = 80.4%, PPV = 19.2%, and NPV = 89.3%; Supplementary Figure 3A). For women, the optimal cutoff value was 22.3 kg (sensitivity = 49.8%, specificity = 56.7%, PPV = 15.9%, and NPV = 87.3%; Supplementary Figure 3B). Baseline on these HGS cutoff values, a binary HGS variable (low vs. normal) was defined for future statistical analysis. There were 1,173 participants (32.1%) classified as having a low HGS based on the cutoff values.

### Relationship between the handgrip strength category and baseline characteristics

The baseline characteristics of participants stratified by the HGS categories are presented in Table 1. Compared to the normal HGS group, the low HGS group was associated with higher age and unmarried rates. In contrast, it was associated with lower proportions of male participants, income, education level, BMI, drinking, and smoking (all *P* < 0.05).

### Multivariable logistic regression analysis

The results of the multivariable logistic regression model analyzing the associations between the HGS and the LSS decline are shown in Table 2. In the fully adjusted model (model 3), continuous HGS was independently associated with reduced odds of LSS decline in the overall population (OR = 0.974, 95% CI = 0.962–0.987) and in men (OR = 0.968, 95% CI = 0.952–0.985), but not in women (OR = 0.983, 95% CI = 0.963–1.003). Similarly, when the HGS was analyzed following separation by 1 standard deviation, consistent results were observed in the overall population (OR = 0.774, 95% CI = 0.680–0.880) and men (OR = 0.756, 95% CI = 0.651–0.878), but not women (OR = 0.880, 0.755–1.019). When dichotomizing HGS based on sex-specific thresholds, participants in the low HGS group had increased odds of experiencing LSS decline compared to those in the normal HGS group (OR = 1.509, 95% CI = 1.218–1.867).

**TABLE 2:** Multivariable models of the relationship between handgrip strength and declined life satisfaction.

	OR (95% CI)				
**Models**	**No./events**	**Model 0^a^**	**Model 1^b^**	**Model 2^c^**	**Model 3^d^**
HGS, continuous	3,649/485	0.978 (0.968–0.987)	0.968 (0.956–0.981)	0.971 (0.959–0.984)	0.974 (0.962–0.987)
Men	1,887/236	0.967 (0.952–0.982)	0.962 (0.946–0.978)	0.965 (0.948–0.981)	0.968 (0.952–0.985)
Women	1,762/249	0.983 (0.964–1.001)	0.978 (0.958–0.997)	0.981 (0.962–1.001)	0.983 (0.963–1.003)
HGS, per 1 SD^e^	3,649/485	0.800 (0.724–0.883)	0.728 (0.641–0.825)	0.751 (0.661–0.853)	0.774 (0.680–0.880)
Men	1,887/236	0.746 (0.650–0.855)	0.714 (0.618–0.825)	0.731 (0.631–0.847)	0.756 (0.651–0.878)
Women	1,762/249	0.878 (0.760–1.009)	0.844 (0.725–0.978)	0.867 (0.744–1.005)	0.880 (0.755–1.019)
HGS, low vs. normal^f^	3,649/485	1.564 (1.285–1.902)	1.651 (1.337–2.036)	1.576 (1.274–1.947)	1.509 (1.218–1.867)

OR (95% CI), odds ratio (95% confidence interval); HGS, handgrip strength; SD, standard deviation.

^a^Model 0 is the unadjusted crude model.

^b^Model 1 is adjusted for the age at baseline (continuous) and sex (reference = female).

^c^Model 2 is adjusted for the age at baseline (continuous), sex (reference = female), body mass index (continuous), education level (reference = lower), hypertension (reference = no), diabetes (reference = no), and heart disease (reference = no).

^d^Model 3 is adjusted for all variables in model 2, plus alcohol drinking (reference = no), smoking (reference = no), residency (reference = dwell in urban community), total household income (continuous), and marital status (reference = married).

^e^Standard deviations of HGS: overall = 9.7 kg, men = 8.7 kg, women = 7.5 kg.

^f^HGS low, men < 27.5 kg or women < 22.3 kg.

### Subgroup and interaction analysis

**FIGURE 5:**
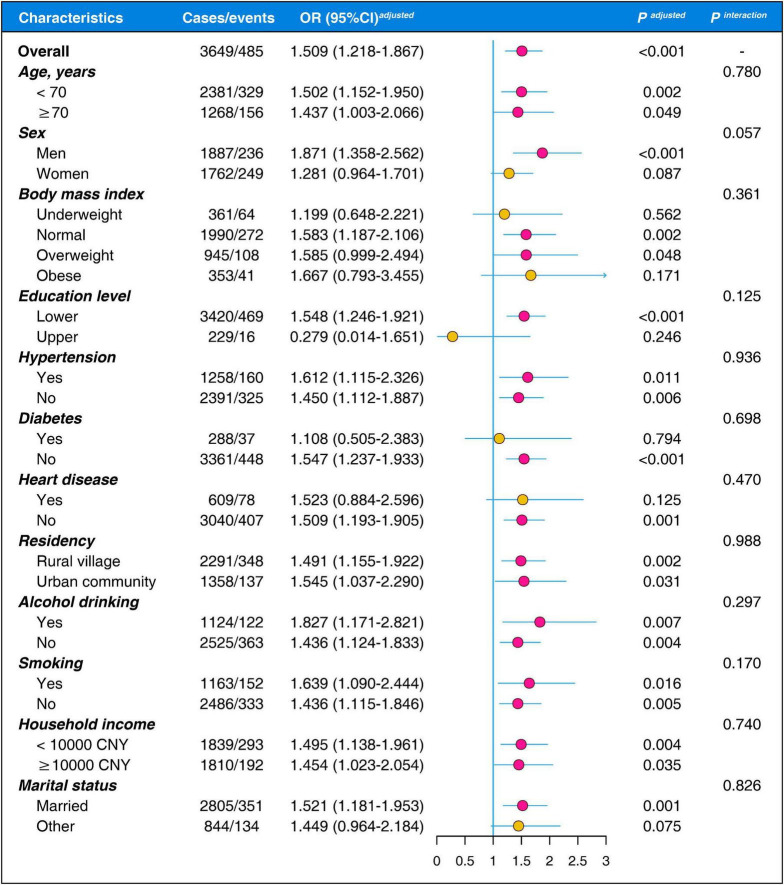
Subgroup and interaction analysis on the associations of low handgrip strength and decline of life satisfaction score. OR, odds ratio; CI, confidence interval; CNY, Chinese Yuan.

The fully adjusted models were repeated in different covariate subgroups to investigate potential effect modifications, and all covariates were screened for potential interactive effects (Figure 5). Consistent with previous findings, the positive association between low HGS and LSS decline was only significant in men (OR = 1.871, 95% CI = 1.358–2.562, *P* < 0.001), but attenuated in women (OR = 1.281, 95% CI = 0.964–1.701, *P* = 0.087, *P* for interaction = 0.057). Additionally, although the observed association between low HGS and LSS decline appeared to be attenuated or modified in participants classified as underweight (OR = 1.199, 95% CI = 0.648–2.221), obese (OR = 1.667, 95% CI = 0.793–3.455), having a higher education level (OR = 0.297, 95% CI = 0.014–1.651), diabetes (OR = 1.108, 95% CI = 0.505–2.383), or other marital status (OR = 1.449, 95% CI = 0.964–2.184), none of these interactions reached statistical significance (all *P* > 0.10).

### Exploratory multivariate analysis

After adjusting for physical activity and sleep duration in multivariate logistic regression models, continuous HGS was independently associated with reduced odds of LSS decline in the overall population (OR = 0.971, 95% CI = 0.951–0.991), in men (OR = 0.973, 95% CI = 0.948–0.999), and in women (OR = 0.962, 95% CI = 0.929–0.994). Similar results were observed when HGS was analyzed by separating values based on 1 standard deviation. Additionally, low HGS, based on sex-specific thresholds, was associated with higher odds of LSS decline (OR = 1.693, 95% CI = 1.175–2.484; Supplementary Table 1).

## Discussion

In this report, we have addressed several knowledge gaps about HGS and LSS decline among older Chinese adults dwelling in the community. Our analysis utilized data from a multicenter, nationally representative survey with standardized data collection. We established sex-specific HGS cutoff values to aid in identifying handgrip weakness associated with LSS. Our study demonstrates that low HGS was independently associated with LSS decline in a dose-dependent linear manner. Subgroup analysis revealed that this association was slightly stronger in men but weakened in women. Further exploratory multivariate analysis showed that, after adjusting for physical performance and sleep duration, the association between HGS and LSS remained consistent in both men and women. Given that handgrip weakness in adults could potentially be a modifiable risk factor, our findings highlight the potential of HGS as a cost-effective indicator of LSS decline. This could, in turn, facilitate the development of intervention strategies to mitigate this decline in older adults.

The existing evidence on the relationship between HGS and LSS is limited (32, 33). Recent research has found that HGS can predict knee pain and osteoarthritis, both of which are associated with disability in older adults (34). Similarly, a study conducted in Brazil demonstrated that low HGS, indicative of functional impairment, was linked to lower life satisfaction (33). Furthermore, a study involving 40,000 participants from the UK Biobank revealed that higher HGS was associated with better cognitive functioning, increased life satisfaction, enhanced subjective wellbeing, and reduced symptoms of depression and anxiety (32). Additionally, low HGS was found to be associated with impaired instrumental activities of daily living (35). Our findings in the present study align with previous studies and provide further support for the relationship between HGS and LSS. Going beyond limited populations or statistical approaches, we aimed to expand on the existing research by investigating the potential nonlinear relationship between HGS and LSS. Moreover, a unique aspect of our study was the derivation of sex-specific cutoff values for HGS, making our results more applicable in public health and clinical settings. It is worth noting that the HGS cutoff value for men (<27.5 kg) established in our study closely aligns with the international consensus proposed by the Asian Working Group for Sarcopenia (<28 kg). This consensus was based on normative HGS data from 26,344 older adults across eight cohorts in Asia (36). The consistency between our findings and this international consensus further enhances the robustness and generalizability of our results across a wider range of applications.

The self-reported nature of the LSS suggests that physical and cognitive dimensions could be two crucial factors influencing the scoring outcomes of the LSS (32, 37). Additionally, sex variances and ethnic disparities may also contribute to these results (32). Recent research has indicated a link between HGS and the physical and mental component summary scores of QoL in patients (38). However, a large-scale study based on the UK Biobank database found that associations between HGS and mental health outcomes were stronger in women than in men (32). In another study involving Asian populations, it was discovered that low HGS was positively correlated with a decline in QoL, as measured by the European Quality of Life Scale-Five Dimensions (EQ-5D) scale (39). However, in our current investigation, the identified connection between HGS and LSS appeared to be slightly strengthened in men, but attenuated in women, suggesting the existence of alternative pathways beyond cognitive function.

While some studies have shown sex differences concerning life satisfaction, it is noteworthy that sex itself does not directly impact life satisfaction. Instead, sex-related physical and social circumstances likely play a significant role in this disparity. Among older women, factors such as chronic health conditions, memory function, self-perceived health status, social support, and social relationships emerge as critical aspects influencing life satisfaction. On the other hand, income and family relationships are highlighted as primary factors influencing satisfaction levels among older men (33, 40). An alternate explanation for the differing effect modifications observed in men and women could stem from variations in the definitions of life satisfaction. While there are resemblances in the content of the evaluations, the definitions of LSS and QoL are not strictly equivalent. Additionally, even within LSS assessments, the use of different evaluation methods may influence the observed associations. For instance, the UK Biobank study utilized a six-item scale to evaluate LSS, covering aspects such as health, family relationships, friendships, financial situation, work/job, and happiness (32). In contrast, the CHARLS employs an overall score to assess LSS. Future research endeavors should aim to provide more precise definitions for the various dimensions of LSS and carry out independent subgroup analyses to delve deeper into uncovering the genuine factors contributing to the decline in LSS associated with low HGS. Furthermore, the development of a standardized LSS scale with global acceptance could potentially facilitate parallel comparisons and data aggregation in future research, offering valuable insights.

The mechanism underlying the results of this study is worth commenting. HGS is an established indicator of skeletal muscle mass and function (10, 41). Skeletal muscles can enhance the upregulation of antioxidant defense systems (42) and possess anti-inflammatory properties (43). Accelerated sarcopenia is observed in transgenic mice lacking the antioxidant enzyme superoxide dismutase 1 (SOD1), which are characterized by diminished mitochondrial bioenergetic function (44). Similarly, mitochondrial respiratory activity in human skeletal muscle has been shown to decrease with advancing age (45). These findings suggest that sarcopenia, as represented by HGS, may be linked to increased systemic inflammatory responses, which can interact with mitochondrial DNA to predict the risk of psychiatric disorders (46) and other aging-induced declines in LSS (47). Additionally, previous evidence shows that sarcopenia-related traits, including lean muscle mass and HGS, have causal effects on brain cortical structure (48). For example, the thickness of the temporal pole and pars triangularis was positively correlated with HGS, indicating that HGS may influence life satisfaction through its regulation of a muscle-brain neurocognitive axis. Furthermore, HGS is related to the epigenetic clock, a biomarker used to estimate biological age based on DNA methylation patterns (47). Individuals with greater grip strength tend to experience a slower rate of epigenetic aging, suggesting that grip strength may serve as a potential predictor of aging-related life satisfaction.

The predominant strength of this study is that it utilizes data from a multicenter, nationally representative survey with standardized data collection. Additionally, it has addressed several knowledge gaps about HGS and LSS decline among older Chinese adults, such as the sex-specific cutoff values in identifying handgrip weakness associated with LSS and the potential nonlinear association between HGS and LSS. However, there are several limitations associated with the present study that need to be acknowledged. Firstly, limited to the original design of the CHARLS project, the estimation of LSS relied on a self-reported overall score. Future studies utilizing a life satisfaction scale with components that reflect the various dimensions of subjects could offer deeper insights. Nonetheless, the simplicity and cost-effectiveness of the LSS assessment employed in this study allow for planned, repeated assessments to capture dynamic changes in life satisfaction status, facilitating the implementation of operational surveillance algorithms. Secondly, given the observational nature of this study, the observed associations do not necessarily imply causation due to potential unmeasured confounding effects and the risk of reverse causality. However, we adjusted for covariates encompassing demographic, lifestyle, comorbidity, and financial status data. Sensitivity analyses were also conducted across different dimensions, such as HGS format, number of covariates, and individual subgroups, to mitigate the risk of reverse causality and enhance the reliability of the observed associations. Nevertheless, future studies adopting a prospective design that assesses the impact of HGS dynamics on LSS are warranted to provide further insights. Thirdly, the HGS cutoffs were determined using the study population and were not validated for efficacy in an external independent dataset. Nonetheless, CHARLS is a nationally representative survey that includes participants reflecting diverse factors (e.g., geographical and economic) in China. Hence, the cutoffs derived from this dataset theoretically possess good representativeness and generalizability. Fourthly, our results are based on data from older adults. Future studies should clarify whether these findings can be replicated in other age groups. Additionally, due to the design of the CHARLS project, dietary survey-related factors were not included in our analysis. Future studies should investigate whether these factors modify the associations we observed. Despite these limitations, the current study offers novel insights into the relationship between HGS and life satisfaction among older adults. It may also pave the way for future research in various directions, such as identifying optimal methods to evaluate life satisfaction and establishing novel HGS cutoff values for detecting LSS decline in other populations. Prospective studies with larger sample sizes encompassing a broader spectrum of ethnic groups will be crucial to address these inquiries effectively.

## Conclusion

This study reveals a positive, linear-like association between low HGS and LSS decline in older Chinese adults. We have also established sex-specific HGS cutoff values according to LSS decline. Additionally, we have observed that the association between handgrip weakness and LSS decline is influenced by sex, with a slightly stronger effect in men and a mitigated effect in women. These findings hold significant implications for public health practitioners and clinicians, offering valuable insights to inform decision-making regarding wellbeing maintenance and the development of innovative strategies to prevent age-related health impairments.

## Data Availability

Publicly available datasets were analyzed in this study. This data can be found here: http://charls.pku.edu.cn/.

## Supplementary material

Supplementary Figure 1 A flowchart of the subject inclusion for exploratory analysis.

Supplementary Figure 2 Comparison of predictive performance between handgrip strength (HGS), body mass index-adjusted handgrip strength (HGS/BMI), and weight-adjusted handgrip strength (HGS/weight). AUC, area under the curve. **(A)** Analysis in men. **(B)** Analysis in women.

Supplementary Figure 3 Receiver operating characteristic (ROC) analyses on the association between handgrip strength and life satisfaction score. AUC, area under the curve. **(A)** ROC analysis in men. **(B)** ROC analysis in women.

Supplementary Table 1 Exploratory analysis of the relationship between handgrip strength and decline in life satisfaction.

## References

[1] Song Y Liu Y Talarico F Zhang Y Hayward J Wang M Associations between differential aging and lifestyle, environment, current, and future health conditions: Findings from Canadian longitudinal study on aging. *Gerontology.* (2023) 69:1394–403. 10.1159/000534015 37725932

[2] Guo L An L Luo F Yu B. Social isolation, loneliness and functional disability in Chinese older women and men: A longitudinal study. *Age Ageing.* (2021) 50:1222–8. 10.1093/ageing/afaa271 33352582

[3] Rantanen T Masaki K Foley D Izmirlian G White L Guralnik J. Grip strength changes over 27 yr in Japanese-American men. *J Appl Physiol.* (1998) 85:2047–53. 10.1152/jappl.1998.85.6.2047 9843525

[4] Gram B Holtermann A Sogaard K Sjogaard G. Effect of individualized worksite exercise training on aerobic capacity and muscle strength among construction workers–A randomized controlled intervention study. *Scand J Work Environ Health.* (2012) 38:467–75. 10.5271/sjweh.3260 22057836

[5] Weiner D Liu C Miao S Fielding R Katzel L Giffuni J Effect of long-term exercise training on physical performance and cardiorespiratory function in adults with CKD: A randomized controlled trial. *Am J Kidney Dis.* (2023) 81:59–66. 10.1053/j.ajkd.2022.06.008 35944747 PMC9780154

[6] Kim M Shinkai S. Prevalence of muscle weakness based on different diagnostic criteria in community-dwelling older adults: A comparison of grip strength dynamometers. *Geriatrics Gerontol Int.* (2017) 17:2089–95. 10.1111/ggi.13027 28517036

[7] Yin L Zhang L Li N Guo J Liu L Lin X Comparison of the AWGS and optimal stratification-defined handgrip strength thresholds for predicting survival in patients with lung cancer. *Nutrition.* (2021) 90:111258. 10.1016/j.nut.2021.111258 33993045

[8] Yin L Song C Cui J Wang N Fan Y Lin X Low fat mass index outperforms handgrip weakness and GLIM-defined malnutrition in predicting cancer survival: Derivation of cutoff values and joint analysis in an observational cohort. *Clin Nutr.* (2021) 41:153–64. 10.1016/j.clnu.2021.11.026 34883304

[9] Cruz-Jentoft A Bahat G Bauer J Boirie Y Bruyere O Cederholm T Sarcopenia: Revised European consensus on definition and diagnosis. *Age Ageing.* (2019) 48:601. 10.1093/ageing/afz046 31081853 PMC6593317

[10] Chen L Woo J Assantachai P Auyeung T Chou M Iijima K Asian working group for Sarcopenia: 2019 Consensus update on sarcopenia diagnosis and treatment. *J Am Med Dir Assoc.* (2020) 21: 300–7.e2. 10.1016/j.jamda.2019.12.012 32033882

[11] Liu M He P Ye Z Zhang Y Zhou C Yang S Association of handgrip strength and walking pace with incident Parkinson’s disease. *J Cachexia Sarcopenia Muscle.* (2024) 15:198–207. 10.1002/jcsm.13366 37990960 PMC10834345

[12] He P Gan X Ye Z Liu M Zhou C Wu Q Combined handgrip strength and walking pace, genetic susceptibility, and incident hypertension: A prospective study in UK Biobank. *Scand J Med Sci Sports.* (2023) 33:989–99. 10.1111/sms.14336 36775263

[13] Sayer A Syddall H Dennison E Martin H Phillips D Cooper C Grip strength and the metabolic syndrome: Findings from the Hertfordshire Cohort Study. *Monthly J Assoc Phys.* (2007) 100:707–13. 10.1093/qjmed/hcm095 17951315 PMC2292249

[14] Boonpor J Parra-Soto S Petermann-Rocha F Ho F Celis-Morales C Gray S. Combined association of walking pace and grip strength with incident type 2 diabetes. *Scand J Med Sci Sports.* (2022) 32:1356–65. 10.1111/sms.14197 35612725 PMC9544034

[15] Grontved A Hu F. Walking pace and handgrip strength: Simple measures of fitness and mortality risk? *Eur Heart J.* (2017) 38:3241–3. 10.1093/eurheartj/ehx497 29020272

[16] Leong D Teo K Rangarajan S Lopez-Jaramillo P Avezum A Orlandini A Prognostic value of grip strength: Findings from the prospective urban rural epidemiology (PURE) study. *Lancet.* (2015) 386:266–73. 10.1016/S0140-6736(14)62000-6 25982160

[17] Celis-Morales C Welsh P Lyall D Steell L Petermann F Anderson J Associations of grip strength with cardiovascular, respiratory, and cancer outcomes and all cause mortality: Prospective cohort study of half a million UK Biobank participants. *Bmj.* (2018) 361:k1651. 10.1136/bmj.k1651 29739772 PMC5939721

[18] Yates T Zaccardi F Dhalwani N Davies M Bakrania K Celis-Morales C Association of walking pace and handgrip strength with all-cause, cardiovascular, and cancer mortality: A UK Biobank observational study. *Eur Heart J.* (2017) 38:3232–40. 10.1093/eurheartj/ehx449 29020281 PMC5837337

[19] Zhuang C Zhang F Li W Wang K Xu H Song C Associations of low handgrip strength with cancer mortality: A multicentre observational study. *J Cachexia Sarcopenia Muscle.* (2020) 11:1476–86. 10.1002/jcsm.12614 32910535 PMC7749566

[20] Zhao X Zhang H Yu J Liu N. Independent and combined associations of handgrip strength and walking speed with cognitive function in older adults: Evidence from a national cross-sectional study. *Aging Ment Health.* (2024) 28:1659–66. 10.1080/13607863.2024.2360018 38835194

[21] Zhao X Chen S Liu N Hu F Yu J. Handgrip strength is positively associated with successful aging in older adults: A national cross-sectional study in China. *J Affect Disord.* (2023) 333:30–7. 10.1016/j.jad.2023.04.041 37084959

[22] Barbosa M Dos Santos M Leite J Rodrigues V de Pinho N Martucci R. Association between functional aspects and health-related quality of life in patients with colorectal cancer: Can handgrip strength be the measure of choice in clinical practice? *Supportive Care Cancer.* (2023) 31:144. 10.1007/s00520-023-07608-7 36729206

[23] van Heinsbergen M Konsten J Bours M Bouvy N Weijenberg M Janssen-Heijnen M. Preoperative handgrip strength is not associated with complications and health-related quality of life after surgery for colorectal cancer. *Sci Rep.* (2020) 10:13005. 10.1038/s41598-020-69806-1 32747640 PMC7400624

[24] Kim J Kang S Kim D Kang H. Associations of physical activity and handgrip strength with health-related quality of life in older Korean cancer survivors. *Cancers.* (2022) 14:6067. 10.3390/cancers14246067 36551553 PMC9776490

[25] Qaisar R Hussain M Karim A Ahmad F Franzese F Al-Masri A The quality of life in Alzheimer’s disease is not associated with handgrip strength but with activities of daily living-a composite study from 28 European countries. *BMC Geriatr.* (2023) 23:536. 10.1186/s12877-023-04233-1 37667196 PMC10478177

[26] Frederiksen H Hjelmborg J Mortensen J McGue M Vaupel J Christensen K. Age trajectories of grip strength: Cross-sectional and longitudinal data among 8,342 Danes aged 46 to 102. *Ann Epidemiol.* (2006) 16:554–62. 10.1016/j.annepidem.2005.10.006 16406245

[27] McNicholl T Curtis L Dubin J Mourtzakis M Nasser R Laporte M Handgrip strength predicts length of stay and quality of life in and out of hospital. *Clin Nutr.* (2020) 39:2501–9. 10.1016/j.clnu.2019.11.006 31757485

[28] Zhao Y Hu Y Smith J Strauss J Yang G. Cohort profile: The China health and retirement longitudinal study (CHARLS). *Int J Epidemiol.* (2014) 43:61–8. 10.1093/ije/dys203 23243115 PMC3937970

[29] Lei X Sun X Strauss J Zhao Y Yang G Hu P Health outcomes and socio-economic status among the mid-aged and elderly in China: Evidence from the CHARLS national baseline data. *J Econ Ageing.* (2014) 4:59–73. 10.1016/j.jeoa.2014.10.001 31428556 PMC6699996

[30] Chen C Lu F Department of Disease Control Ministry of Health PR China. The guidelines for prevention and control of overweight and obesity in Chinese adults. *Biomed Environ Sci.* (2004) 17:1–36. 15807475

[31] Xiao J Dong X Ding M Kong T. Adverse childhood experiences, sleep quality/duration and later-life lower extremity function among older adults in China: Evidence from CHARLS. *BMC Psychol.* (2025) 13:73. 10.1186/s40359-025-02396-7 39871330 PMC11773826

[32] Jiang R Westwater M Noble S Rosenblatt M Dai W Qi S Associations between grip strength, brain structure, and mental health in > 40,000 participants from the UK Biobank. *BMC Med.* (2022) 20:286. 10.1186/s12916-022-02490-2 36076200 PMC9461129

[33] Pinto J Neri A. Factors associated with low life life satisfaction in community-dwelling elderly: FIBRA Study. *Cad Saude Publica.* (2013) 29:2447–58. 10.1590/0102-311x00173212 24356691

[34] Tan G Kioh S Mat S Chan S Lee J Tan Y Physical and psychosocial characteristics differ between individuals with knee pain and different knee osteoarthritis diagnostic criteria. *Postgrad Med J.* (2023) 99:1104–9. 10.1093/postmj/qgad049 37392161

[35] Gopinath B Kifley A Liew G Mitchell P. Handgrip strength and its association with functional independence, depressive symptoms and quality of life in older adults. *Maturitas.* (2017) 106:92–4. 10.1016/j.maturitas.2017.09.009 29150172

[36] AuyeungTAraiHChenLWooJ. Letter to the editor: Normative data of handgrip strength in 26344 older adults - A pooled dataset from eight cohorts in Asia. *J Nutr Health Aging.* (2020) 24:125–6. 10.1007/s12603-019-1287-6 31886819

[37] Fritz N McCarthy C Adamo D. Handgrip strength as a means of monitoring progression of cognitive decline - A scoping review. *Ageing Res Rev.* (2017) 35:112–23. 10.1016/j.arr.2017.01.004 28189666

[38] Jakobsen L Rask I Kondrup J. Validation of handgrip strength and endurance as a measure of physical function and quality of life in healthy subjects and patients. *Nutrition.* (2010) 26:542–50. 10.1016/j.nut.2009.06.015 19804953

[39] Kang S Lim J Park H. Relationship between low handgrip strength and quality of life in Korean men and women. *Q Res.* (2018) 27:2571–80. 10.1007/s11136-018-1920-6 29922911

[40] Oshio T. Gender differences in the associations of life satisfaction with family and social relations among the Japanese elderly. *J Cross Cult Gerontol.* (2012) 27:259–74. 10.1007/s10823-012-9169-y 22648323

[41] Kirk B Cawthon P Arai H Avila-Funes J Barazzoni R Bhasin S The Conceptual definition of sarcopenia: Delphi consensus from the global leadership initiative in sarcopenia (GLIS). *Age Ageing.* (2024) 53:afae052. 10.1093/ageing/afae052 38520141 PMC10960072

[42] Ji L. Antioxidant signaling in skeletal muscle: A brief review. *Exp Gerontol.* (2007) 42:582–93. 10.1016/j.exger.2007.03.002 17467943

[43] Nielsen S Pedersen B. Skeletal muscle as an immunogenic organ. *Curr Opin Pharmacol.* (2008) 8:346–51. 10.1016/j.coph.2008.02.005 18417420

[44] Jang Y Lustgarten M Liu Y Muller F Bhattacharya A Liang H Increased superoxide in vivo accelerates age-associated muscle atrophy through mitochondrial dysfunction and neuromuscular junction degeneration. *FASEB J.* (2010) 24:1376–90. 10.1096/fj.09-146308 20040516 PMC2987499

[45] Short K Bigelow M Kahl J Singh R Coenen-Schimke J Raghavakaimal S Decline in skeletal muscle mitochondrial function with aging in humans. *Proc Natl Acad Sci U S Am.* (2005) 102:5618–23. 10.1073/pnas.0501559102 15800038 PMC556267

[46] Liu L Cheng S Qi X Meng P Yang X Pan C Mitochondria-wide association study observed significant interactions of mitochondrial respiratory and the inflammatory in the development of anxiety and depression. *Transl Psychiatry.* (2023) 13:216. 10.1038/s41398-023-02518-y 37344456 PMC10284875

[47] Chen L. The grip on healthspan: Handgrip strength as a vital sign of aging. *Arch Gerontol Geriatr.* (2024) 122:105436. 10.1016/j.archger.2024.105436 38584043

[48] Zhan Y Zhang Z Lin S Du B Zhang K Wu J Causal association of sarcopenia-related traits with brain cortical structure: A bidirectional Mendelian randomization study. *Aging Clin Exp Res.* (2025) 37:57. 10.1007/s40520-025-02977-x 40014117 PMC11868162

