# Supplementary material for: Handgrip weakness and overall life satisfaction decline: derivation of cutoff values and analysis of sex differences in older Chinese adults
Source: Front Nutr. 2025 Mar 18;12:1537818. doi: 10.3389/fnut.2025.1537818 (PMC11958193; doi:10.3389/fnut.2025.1537818)
Supplement: Supplementary Figure 1 — A flowchart of the subject inclusion for exploratory analysis. [file Data_Sheet_1.pdf]

Figure S1

17708 community-dwelling participants assessed

→ **10418** participants aged < 60 years excluded

7290 potentially eligible participants

→ **3587** participants exclued due to missing values on any study variable

- **1476** on body height
- **1460** on body weight
- **1509** on body mass index
- **3** on education levels
- **83** on hypertension
- **112** on diabetes
- **98** on heart disease
- **61** on alcohol drinking status
- **255** on smoking status
- **1550** on handgrip strength
- **1882** on total household income
- **1446** on life satisfaction score
- **9** on marital status

*An individual may have missing values for multiple variables

3703 potentially eligible participants

→ **54** participants excluded due to outlier values

- **1** participant with body mass index = 0
- **53** participants with handgrip strength = 0

3649 participants included for analysis

→ **2098** participants excluded due to outlier values

- **2082** on vigorous energetic physical activity
- **2082** on moderate energetic physical activity
- **2086** on mild energetic physical activity
- **25** on sleep duration

*An individual may have missing values for multiple variables

1551 participants for exploratory analysis

Figure S2

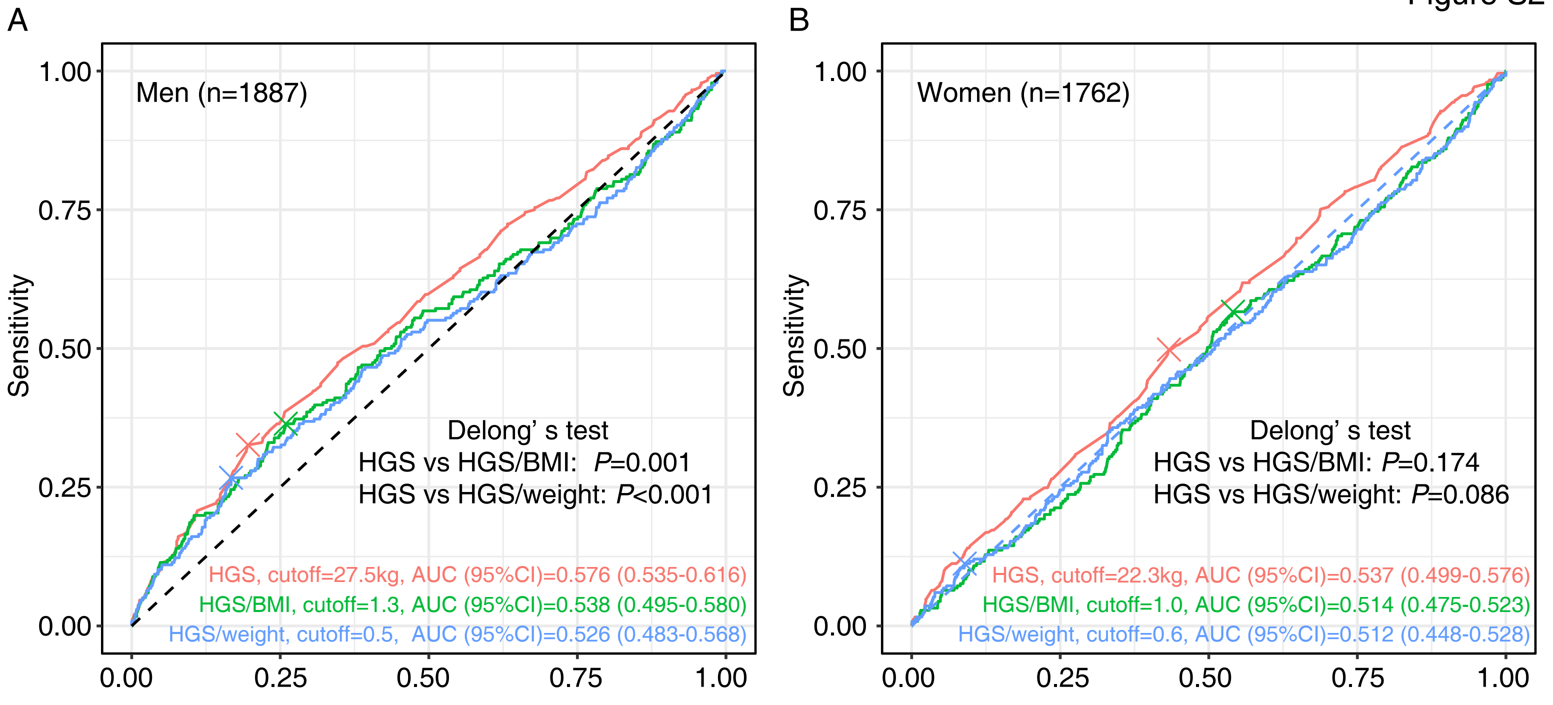
A
Men (n=1887)
Sensitivity
1.00
0.75
0.50
0.25
0.00
0.00
0.25
0.50
0.75
1.00
Delong' s test
HGS vs HGS/BMI: P=0.001
HGS vs HGS/weight: P<0.001
HGS, cutoff=27.5kg, AUC (95%CI)=0.576 (0.535-0.616)
HGS/BMI, cutoff=1.3, AUC (95%CI)=0.538 (0.495-0.580)
HGS/weight, cutoff=0.5, AUC (95%CI)=0.526 (0.483-0.568)
B
Women (n=1762)
Sensitivity
1.00
0.75
0.50
0.25
0.00
0.00
0.25
0.50
0.75
1.00
Delong' s test
HGS vs HGS/BMI: P=0.174
HGS vs HGS/weight: P=0.086
HGS, cutoff=22.3kg, AUC (95%CI)=0.537 (0.499-0.576)
HGS/BMI, cutoff=1.0, AUC (95%CI)=0.514 (0.475-0.523)
HGS/weight, cutoff=0.6, AUC (95%CI)=0.512 (0.448-0.528)

Figure S3

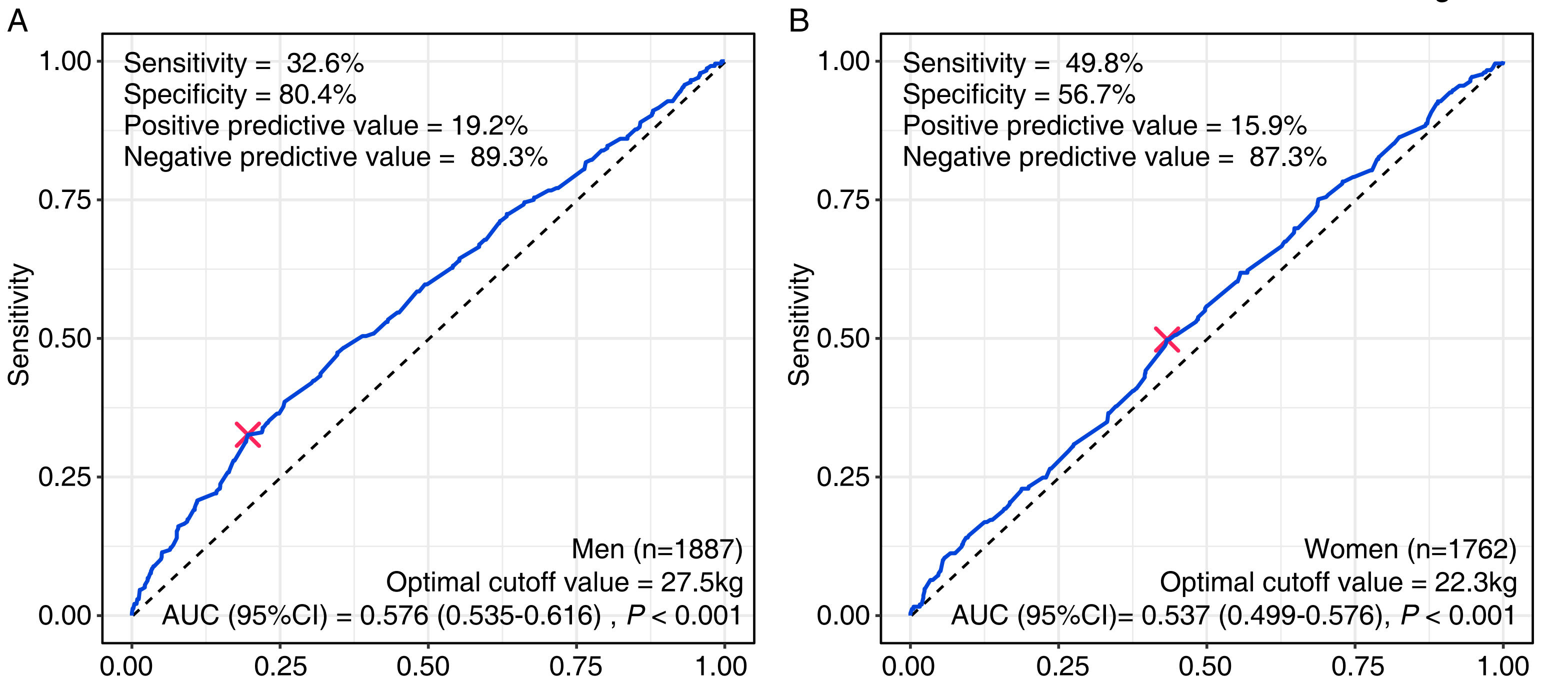
A
Sensitivity = 32.6%
Specificity = 80.4%
Positive predictive value = 19.2%
Negative predictive value = 89.3%
Men (n=1887)
Optimal cutoff value = 27.5kg
AUC (95%CI) = 0.576 (0.535-0.616) , P < 0.001
Sensitivity
1.00
0.75
0.50
0.25
0.00
0.00
0.25
0.50
0.75
1.00
B
Sensitivity = 49.8%
Specificity = 56.7%
Positive predictive value = 15.9%
Negative predictive value = 87.3%
Women (n=1762)
Optimal cutoff value = 22.3kg
AUC (95%CI)= 0.537 (0.499-0.576), P < 0.001
Sensitivity

Table S1. Exploratory analysis of the relationship between handgrip strength and decline in life satisfaction

Models	No./events	Adjusted OR (95%CI)[a]
HGS, continuous	1551/201	0.971 (0.951-0.991)
Men	792/98	0.973 (0.948-0.999)
Women	759/103	0.962 (0.929-0.994)
HGS, per 1 SD[b]	1551/201	0.750 (0.616-0.913)
Men	792/98	0.787 (0.625-0.992)
Women	759/103	0.757 (0.592-0.956)
HGS, low vs. normal	1551/201	1.693 (1.175-2.484)

Abbreviations: OR (95%CI), hazard ratio (95% confidence interval); HGS, handgrip strength; SD, standard deviation.

[a] Model is adjusted for the age at baseline (continuous), sex (reference=female), body mass index (continuous), education level (reference=lower), hypertension (reference=no), diabetes (reference=no) and heart disease (reference=no), alcohol drinking (reference=no), smoking (reference=no), residency (reference=dwell in urban community), total household income (continuous), marital status (reference=married), vigorous energetic physical activity at least 10 minutes every week (reference=no), moderate energetic physical activity at least 10 minutes every week (reference=no), mild energetic physical activity at least 10 minutes every week (reference=no), and sleep duration (average hours for one night sleeping time during the past month, continuous).

[b] Standard deviations of HGS: overall=9.7kg, men=8.9kg, women =7.2kg.
